# A novel index using optical coherence tomography for predicting anatomical and visual outcomes of idiopathic macular hole surgery

**DOI:** 10.1186/s40942-026-00817-2

**Published:** 2026-02-20

**Authors:** Mehrdad Motamed Shariati, Seyedeh Maryam Hosseini, Naser Shoeibi, Ali Motamed Shariati, Mohammadreza Ansari Astaneh, Ghodsieh Zamani, Mojtaba Abrishami, Majid Abrishami, Farzaneh Samimi-nia, Zahra Moravvej

**Affiliations:** Eye Research Center, Mashhad University of Medical Sciences, Mashhad, Iran

**Keywords:** Idiopathic full thickness macular hole, Visual outcome, Vitrectomy, Optical coherence tomography

## Abstract

**Background:**

In this study, we introduced a new optical coherence tomography index, Macular Hole Reflectivity Index (MHRI), for macular hole surgery prognosis. Visual acuity improvement is unpredictable in macular hole surgery, and as the reflectivity of RPE can be a biomarker for the status of RPE, we aimed to evaluate the new index in terms of anatomical and visual outcomes.

**Methods:**

A retrospective consecutive case series of 48 patients with idiopathic full-thickness macular hole (FTMH) who had undergone pars plana vitrectomy with inverted internal limiting membrane (ILM) flap was performed. To calculate the MHRI, the spectral domain optic coherence tomography (SD-OCT) images were converted into 8-bit greyscale images. The MHRI was defined as the ratio of Maximum central reflectivity / Minimum paracentral reflectivity. Analysis was performed to assess if the index was an independent predictor of post-operative VA, and the MHRI cutoffs were calculated based on the receiver operating characteristic curve (AUC) to evaluate the ability of indices to predict surgical and visual success.

**Results:**

The MHRI was significantly lower in the surgical success group (*p* < 0.0001). MHRI was significantly correlated with the postoperative VA. MHRI exhibited an AUC value of 97.0%, and the cutoff value of 1.65 demonstrated a sensitivity of 91% and a specificity of 100% for anatomical closure. The prognostic value of MHRI was further evaluated by analysis of those with successful surgical closure for visual success. The AUC for predicting visual success after FTMH surgery was 76.1%, with a cutoff value of 1.38.

**Conclusion:**

MHRI demonstrated to be an efficient index in the prediction of anatomical and visual success in macular hole surgery. However, larger multicenter studies are required to validate the applicability and predictive value of this index in clinical settings.

## Background

Idiopathic full-thickness macular hole (FTMH) is a defect of the neurosensory retina at the fovea, which causes significant visual impairment [1, 2]. The prevalence of idiopathic FTMH is reported to be 0.2 to 3.3 per 1000 adults and occurs in both eyes in up to 15.6% of cases [3–6]. Since the first report of FTMH treatment with vitrectomy in 1991 [7], vitrectomy and internal limiting membrane (ILM) peeling alongside several adjunctive techniques have become the mainstay of treatment [8–10].

In recent studies the anatomical closure rate following primary surgery varies from 80 to 97% [11–14]. It is known that hole size is one of the most important factors for surgical success regarding hole closure, and success declines for larger holes. Two studies to date have shown a reduction in success rates for holes exceeding 500 μm in minimum hole diameter [15, 16].

Despite the high surgical closure rates, visual acuity (VA) improvement is unpredictable, and VA greater than 20/40 is achieved in approximately 50% of the cases [17]. Researchers have attempted to identify the prognostic factors for postoperative visual gain. Several factors, including preoperative VA, ELM defect, minimum hole diameter, duration of macular hole, and base diameter have been found to be related to the postoperative VA [18–20]. Optical coherence tomography (OCT), which provides high-resolution visualization of the retinal microstructure, has been widely used for the diagnosis and prognosis of FTMH [21]. So far, several OCT-based parameters and indices have been introduced to predict the surgical success and visual outcome of FTMH, including macular hole index (MHI), diameter hole index (DHI), tractional hole index (THI), and hole form factor (HFF) [18, 22–24].

The reflectivity of the retinal pigment epithelium (RPE) on OCT is primarily influenced by the presence and distribution of intracellular organelles, particularly melanin and mitochondria. In healthy eyes, the RPE appears as a broad, highly reflective band due to the high concentration of these light-scattering components [25]. Reduced RPE reflectivity can signify RPE atrophy or degeneration, where there is a decrease in melanin granules and RPE cell density. This thinning or loss of RPE allows for greater light penetration to deeper structures, sometimes leading to an apparent increase in the reflectivity of the choroid beneath. Conversely, increased or altered RPE reflectivity can be caused by the accumulation of lipofuscin and melanolipofuscin within RPE cells, which are byproducts of photoreceptor outer segment phagocytosis. RPE migration and cellular dysmorphia can also lead to changes in reflectivity patterns [26].

As the reflectivity of RPE can be a biomarker for the status of RPE, in the present study we aim to introduce the new Macular Hole Reflectivity Index (MHRI) (maximum central reflectivity / minimum paracentral reflectivity). To identify the prognostic value of this index in hole closure and the visual outcome in those that close, we examined the visual and anatomical outcomes in a retrospective study of 48 FTMHs treated by vitrectomy with an inverted ILM flap technique.

## Methods

### Patients

This study is a retrospective interventional case series of patients who were operated on for idiopathic FTMH between 2021 and 2024. This study was approved by the Regional Ethical Committee of Mashhad University of Medical Sciences and adheres to the tenets of the Declaration of Helsinki.

Patients who were referred to our retina clinic with an idiopathic full thickness macular hole were included. Patients with lamellar macular holes, posttraumatic macular holes and/or any other retinal disorders or prior retinal surgery were excluded from the study. Informed consent was obtained from all patients enrolled.

Records of 48 patients with FTMH undergoing macular hole surgery with at least three months follow-up were studied. Demographic data of patients including gender, age, pre- and post-operative best corrected visual acuity (BCVA) were recorded in Snellen and converted to a logMAR equivalent. The last recorded post-operative VA in follow-up was used for comparison. Patients underwent thorough ophthalmic examinations, including slit-lamp biomicroscopy, fundoscopy, and SD-OCT before surgery and after surgery at 3-month visit.

### OCT parameter measurement

OCT examinations were acquired with Spectralis SD-OCT (Heidelberg Engineering, Heidelberg.

Germany) by experienced technicians. A pattern size of 20°×20° was used to acquire 25 B-scan images, each composed of 512 A-scans. A horizontal raster scan was obtained and aligned along the fovea-to-disc axis. Images with low-quality signals (< 60) and artifacts were excluded. All OCT images were marked twice by each rater at least 4 weeks apart to test for intra- and interobserver agreement.

All OCT imaging evaluations and measurements were performed by two retina specialists (authors M.H. and N.S.). The retinal specialists were uninformed of the postoperative results when the measurements were performed.

**Fig. 1:**
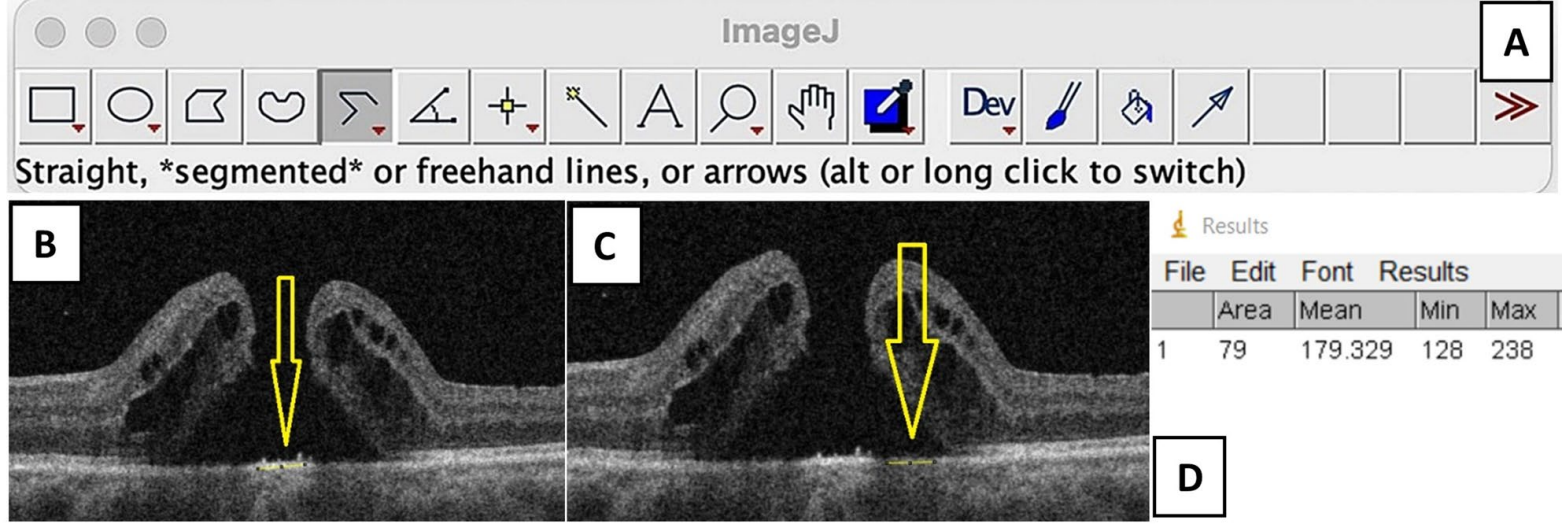
(**A**) The “straight” line mode of the Image J software (**B**) The straight line is drawn along the central hyperreflective region of the macular hole (yellow line shown with a yellow arrow) (**C**) The straight line is drawn along the paracentral hypo-reflective region of the macular hole (yellow line shown with a yellow arrow) (**D**) The mean, minimum and maximum reflectivity of the line

The preoperative minimum linear diameter *(MLD)* of the macular hole, defined as the minimum distance between the inner edges of the MH, was manually measured using ImageJ software (version 1.8.0). To calculate the MHRI, OCT images were first exported and converted into 8-bit greyscale images. Images were then processed using the “straight” line option from the toolbar (Fig. 1A). The mean reflectivity (brightness) of the normal retinal pigmented epithelium (RPE)/Bruch’s membrane complex was measured. The same method was used to obtain the maximum and mean reflectivity at the central hyperreflective region of the macular hole and also the minimum and mean reflectivity at the paracentral hyporeflective regions on either side of the central hyperreflective region (Fig. 1B-D). After measuring these parameters, five indices were derived, which are depicted in Table 1. The MHRI was defined as the ratio of Maximum central reflectivity / Minimum paracentral reflectivity.

**Table 1:** Definition of the introduced OCT indices

Index	Definition (formula)
CRI-Max	Maximum central brightness/Normal RPE brightness
CRI-Mean	Mean central brightness/Normal RPE brightness
PCRI-Min	Minimum paracentral brightness/Normal RPE brightness
PCRI-Mean	Mean paracentral brightness/Normal RPE brightness
MHRI	Maximum central brightness/Minimum paracentral brightness

CRI-Max: Central reflectivity index-maximum, CRI-Mean: Central reflectivity index-mean, PCRI-Min: Paracentral reflectivity index-minimum, PCRI-Mean: Paracentral reflectivity index-mean, MHRI: Macular hole reflectivity index

### Surgical details

All surgeries were performed by two highly experienced retinal surgeons (M.H, N.S). The surgical technique was identical for all patients. To minimize any potential bias related to surgical technique, all enrolled patients underwent the same technique regardless of macular hole size. The procedure included standard three-port pars plana vitrectomy with 23 gauge with inverted ILM flap. For posterior vitreous detachment induction, triamcinolone acetate was used as a visualizing adjuvant. MembraneBlue-Dual (DORC, the Netherlands) was applied for 30 s to stain the ILM and assist in visualization. ILM peeling and flap formation were done with the ILM end-gripping forceps. In the end, tamponade was performed with air, and patients were required to hold a face-down position for 7 days as much as possible. In patients with cataract, phacoemulsification with intraocular lens implantation was performed before pars plana vitrectomy.

### Statistical analysis

The normality of data distribution was assessed with Shapiro-Wilk tests. Data for continuous variables are presented as mean ± standard deviation and as frequencies (percentages) for categorical variables. The visual acuity before and after surgery was compared with paired-sample t-test. Normally distributed data were compared using the Student’s t-test, and the Mann–Whitney U test was performed for non-parametric data and categorical data by Fisher’s exact test. Spearman’s correlation was used to investigate correlations. To assess reproducibility (inter-rater agreement), intraclass correlation coefficients (ICCs) were measured, and Bland-Altman analysis were performed for MHRI measurements. Multiple linear regression was performed to assess if the OCT parameter and indices were independent predictors of postoperative VA after controlling for various covariates such as age, surgery type, and preoperative VA. Regression assumptions were formally assessed, multicollinearity was evaluated by examining Variance Inflation Factor (VIF) and tolerance values for all predictors. All VIF values were below 5 indicating that multicollinearity is not a concern in the model, with MLD having VIF = 1.13 and tolerance = 0.88 and MHRI having VIF = 1.06 and tolerance = 0.93. Finally, the MHRI cutoffs were determined based on receiver operating characteristic (ROC) curve analysis to evaluate the ability of indices to predict surgical and visual success. Visual success was defined as postoperative VA ≤ 0.3 LogMAR according to similar MH prognostic studies [15]. Results were considered statistically significant if the *P* value was < 0.05. Statistical analyses were conducted using IBM SPSS Statistics (version 26.0; IBM Corp., Armonk, NY).

## Results

A total of 48 patients were included with a mean age of 67.8 ± 7.7 years, and 60.4% of the patients were female. Of the 48 eyes with macular hole, 10 (20.8%) had previously undergone cataract surgery, and vitrectomy combined with phacoemulsification was carried out in 28 (58.3%) operations. The mean macular hole size was 308.7 ± 76.9 μm with a normal distribution.

There were no sex-dependent differences in the parameters including age, pre- and post-operative VA, MLD, or the closure rate.

Patients were followed up after the operation for a minimum of 3 months, and the mean follow-up duration was 12.27 ± 9.6 months. Anatomical closure of the macular hole was achieved in 45 (93.75%) eyes. No intra- or post-operative complications were noted.

When comparing patients with an anatomical MH closure (surgical closure success group) to those with a persisting MH after surgery (surgical closure failure), there was no difference in sex, age, pre-op VA, and lens status (Table 2). Evidently, the postoperative VA differed significantly between the surgical closure success and failure groups (0.3 ± 0.2 vs. 1.0 + 0.4 LogMAR, respectively; *p* < 0.0001).

**Table 2:** Overview of the evaluated parameters and comparison between the parameters in the surgical closure groups

	Overall *n* = 48	Surgical closure success *n* = 45	Surgical closure failure *n* = 3	*P* value
Age (years)	67.8 ± 7.7	67.6 ± 7.9	72.0 ± 2.6	0.351^$^
Female (%)	29 (60.4)	27 (60)	2 (66.7)	0.657^§^
VA preop (logMAR)	0.9 ± 0.3	0.9 ± 0.3	0.9 ± 0.4	0.801^#^
Pseudophakia (%)	10 (20.8)	9 (20)	1 (33.3)	0.512^§^
MLD (µm)	308.7 ± 76.9	300.4 ± 71.8	433.3 ± 21.3	0.003^#^
Surgery type *Vitx + PE / Vitx*	28 / 20	26 / 19	2 / 1	0.621^§^
CRI-Max	1.06 ± 0.88	1.06 ± 0.86	1.14 ± 0.18	0.151^#^
CRI-Mean	0.98 ± 0.56	0.98 ± 0.05	0.99 ± 0.04	0.543^#^
PCRI-Min	0.74 ± 0.12	0.75 ± 0.11	0.52 ± 0.08	0.001^#^
PCRI-Mean	0.80 ± 0.10	0.81 ± 0.10	0.63 ± 0.03	0.005^#^
MHRI	1.47 ± 0.29	1.42 ± 0.16	2.24 ± 0.69	< 0.0001^#^
VA postop (logMAR)	0.4 ± 0.3	0.3 ± 0.2	1.0 + 0.4	< 0.0001^#^

Statistical tests applied: ^$^ T-test ^#^ Mann–Whitney U test; ^§^ Fisher’s exact test; Abbreviations: MLD: minimum linear diameter, CRI-Max: Central reflectivity index-maximum, CRI-Mean: Central reflectivity index-mean, PCRI-Min: Paracentral reflectivity index-minimum, PCRI-Mean: Paracentral reflectivity index-mean, MHRI: Macular hole reflectivity index

**Fig. 2:**
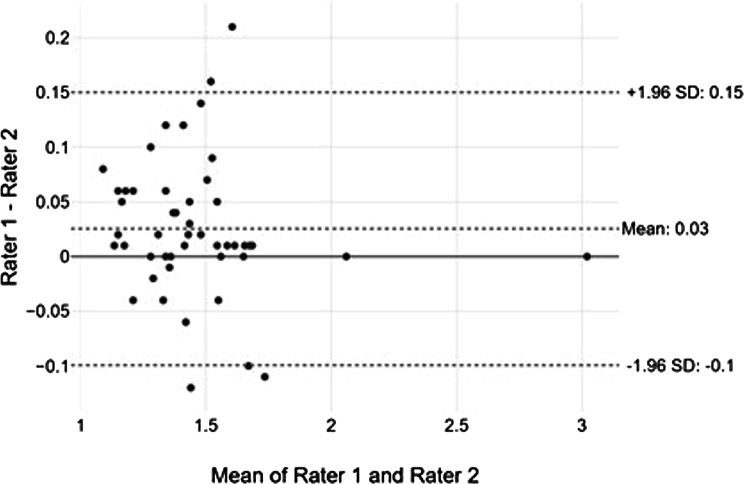
Bland-Altman plot for the inter-rater agreement of MHRI measurements. The x-axis displays the mean MHRI measurements of rater 1 and 2, and the y-axis displays the difference between the measurements of raters

The intraclass correlation coefficients (ICCs) for interobserver agreement between rater 1 and rater 2 were 0.98, 0.99, 0.98, 0.97, and 0.98 for measurement of the maximum central reflectivity, mean central reflectivity, minimum paracentral reflectivity, mean paracentral reflectivity, and normal RPE reflectivity, respectively. Difference between measurements of MHRI to their mean, together with the 95% Limits of Agreement (LOA), is displayed as a Bland-Altman plot (Fig. 2).

The comparison of the OCT parameters revealed a difference between the groups for the MLD. The MLD was smaller in the surgical success group (closure success: 300.4 ± 71.8 μm vs. faliure: 433.3 ± 21.3 μm; *p* = 0.003). Also, the introduced OCT indices including CRI-Max, CRI-Mean, PCRI-Min, PCRI-Mean, and MHRI were compared between the two groups. There was a significant difference in PCRI-Min, PCRI-Mean, and MHRI between the groups (Table 2). The MHRI was lower in the surgical success group (1.42 ± 0.16 vs. 2.24 ± 0.69, *p* < 0.0001).

The mean VA was 0.9 ± 0.3 logMAR prior to surgery, which significantly increased to 0.4 ± 0.3 logMAR postoperatively (*p* < 0.0001). In Spearman’s correlation analysis, preoperative VA showed a significant (*p* = 0.03) correlation with postoperative VA (*r* = 0.305). The results showed no correlation between patients’ age and postoperative VA (*p* = 0.360). Visual success, which was defined as a postoperative vision of 0.3 logMAR or better, was achieved in 26 (57.7%) of the 45 eyes with successful anatomical closure. Visual success was more often achieved for smaller holes (258.6 ± 57.3 vs. 357.5 ± 45.6 μm, *p* < 0.001). No statistically significant differences in the visual success rates were observed in terms of sex, pre-operative VA, and type of surgery (Vitx vs. Vitx + PE).

In correlation analysis, CRI-Max and CRI-Mean did not significantly correlate with postoperative VA. PCRI-Mean, PCRI-Max, and MHRI negatively correlated with postoperative VA (Table 3).

**Table 3:** Correlation analysis of indices with postoperative LogMAR VA

Parameter	Correlation coefficient	*P* value
CRI-Max	0.125	0.399
CRI-Mean	0.208	0.156
PCRI-Min	-0.518	< 0.0001
PCRI-Mean	-0.498	< 0.0001
MHRI	-0.551	< 0.0001
MLD	0.857	< 0.0001

MLD: minimum linear diameter, CRI-Max: Central reflectivity index-maximum, CRI-Mean: Central reflectivity index-mean, PCRI-Min: Paracentral reflectivity index-minimum, PCRI-Mean: Paracentral reflectivity index-mean, MHRI: Macular hole reflectivity index

Multiple regression analysis adjusted for confounding factors showed that among the indices, MLD and MHRI significantly predicted postoperative VA (Table 4). Therefore, a lower minimum linear diameter and a lower MHRI was correlated with a higher postoperative VA. MLD and MHRI showed positive correlation (*r* = 0.447, *p* < 0.001).

**Table 4:** Multiple regression analysis of factors predicting postoperative LogMAR VA (confounding age, preVA, surgery)

Parameter	Coefficient	95% CI	*P* value
MHRI	0.581	0.331 to 0.832	< 0.0001
MLD	0.003	0.003 to 0.004	< 0.0001

MLD: minimum linear diameter, MHRI: Macular hole reflectivity index

**Fig. 3:**
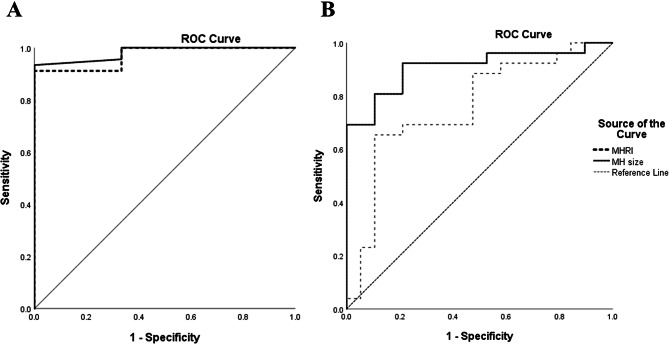
Receiver operating characteristic (ROC) curves for the MLD and MHRI for predicting surgical and visual success. A larger area under the curve represents a higher false-negative rate and a lower false-positive rate and thus a high sensitivity and specificity. (**A**) ROC of MHRI and MLD for anatomical success (**B**) ROC of MHRI and MLD for visual success (VA ≤ 0.3 LogMAR)

The ROC analysis showed that MHRI predicted surgical success relatively well. The area under the curve (AUC) was 97.0% (95% CI; 91% to 100%) (Table 5; Fig. 3).

**Table 5:** Receiver operating characteristic (ROC) curve data and cutoff values for predicting surgical success (Anatomical closure)

Parameter	AUROC	95% CI	Cutoff value	Youden’s index (J)	Sensitivity (%)	Specificity (%)	*P* value
MHRI	0.970	0.911 to 1.000	1.65	0.91	91%	100%	0.007
MLD	0.981	0.940 to 1.000	404.5	0.93	93%	100%	0.006

The cutoff value was derived according to the maximum value of Youden’s index (J). Sensitivity and specificity show the highest possible values at the cutoff value. Abbreviations: CI confidence interval, MLD minimum linear diameter, MHRI: Macular hole reflectivity index

Eligible for visual success ROC analysis were 45 macular hole operations with successful anatomical hole closure. The ROC for MHRI and visual success (VA ≤ 0.3 LogMAR) had an area under the curve of 76.1% (95% CI; 66%–92%) (Table 6).

**Table 6:** Receiver operating characteristic (ROC) curve data and cutoff values for predicting postoperative VA success (post-op VA ≤ 0.3 LogMAR)

Parameter	AUROC	95% CI	Cutoff value	Youden’s index (J)	Sensitivity (%)	Specificity (%)	*P* value
MHRI	0.761	0.614 to 0.908	1.38	0.54	65.4%	89.4%	0.003
MLD	0.909	0.821 to 0.997	313.5	0.71	92.3%	78.9%	< 0.0001

The cutoff value was derived according to the maximum value of Youden’s index (J). Sensitivity and specificity show the highest possible values at the cutoff value. Abbreviations: CI confidence interval, MLD minimum linear diameter, MHRI: Macular hole reflectivity index

## Discussion

Numerous clinical factors and OCT-based parameters have been previously considered for predicting the surgical and visual outcome of FTMH surgery. The first OCT parameter evaluated has been the MLD, which is defined as the shortest distance across the full-thickness defect. Ip et al. indicated that holes smaller than 400 μm demonstrated higher success rates for closure and greater visual acuity improvement [27]. Ruiz-Moreno et al. also showed that macular holes with a minimum length less than 311 μm have a better visual prognosis [24]. In the present study, we demonstrated similar results; according to our ROC curve analysis for MLD, the cutoff value for surgical closure was 404.5 μm, and the cutoff for better visual outcome (post-op VA ≤ 0.3 LogMAR) was 313.5 μm. Steel et al. and Ch’ng et al. proposed MLD of 500 μm and 630 μm respectively, as threshold values for surgical success [12, 15]. The ROC curve for surgical failure and MLD in the study of Steel et al. had an AUROC of 77.9%, while in our study it was 98.1% [15]. Also, Ch’ng et al. reported a Youden index of 0.46 for their cutoff value, which indicates a relatively low to moderate level of effectiveness in contrast to our cutoff with a Youden index of 0.93 [12].

Other indices have also been suggested in literature with variable results and predictive values. The MHI proposed by Kusuhara et al. is another OCT parameter, defined as the ratio between hole height and base diameter; a higher MHI value indicates a smaller horizontal and a greater vertical hole dimension [22]. They concluded that the postoperative BCVA in eyes with MHI ≥ 0.5 was better than those with MHI < 0.5. Several other studies have shown a correlation between MHI and postoperative VA [19, 22, 28–30]. Ruiz-Moreno et al. found that MHI was correlated significantly with postoperative VA, although in ROC curve analysis they showed that MHI had little predictive value [24]. The THI (ratio of height to minimum diameter) has been demonstrated to have correlations with postoperative visual acuity in several studies [19, 24, 28]. Studies evaluating the DHI (ratio of minimum diameter to baseline diameter) have established inconsistent conclusions. Although Chhablani et al. found a significant correlation between DHI and anatomical closure, its correlation with postoperative VA was not confirmed in their study as well as other similar reports [19, 24, 28, 31].

In this study, we introduced a new preoperative OCT index for the prognosis of FTMH surgery.

This new index, which is based on RPE reflectivity, proved to have predictive value for the surgical and functional outcome of FTMH surgery. The MHRI which we proposed as the ratio of maximum central RPE reflectivity to the minimum paracentral RPE reflectivity, was significantly higher in surgically failed macular hole closure eyes. Regarding the diagnostic capability of MHRI, we performed a ROC curve analysis. The ROC curve obtained for MHRI as a predicator of anatomical closure had 100% specificity and 91% sensitivity with a Youden’s index of 0.91 at the cutoff of 1.65. Although as there were only three failures and a relatively small sample size, AUROC and cutoff values should be interpreted with caution, and its discriminative performance should be evaluated in further studies.

When anatomical closure is achieved after surgery, the visual gain depends on the initial photoreceptor damage and the repair processes. As the anatomical closure of the macular hole is not the sole factor impacting the visual outcome of the surgery, we assume that MHRI which may be an indication of RPE and photoreceptor condition, could serve as a valuable index. Similar to previous studies, since our goal was to evaluate this index in terms of visual improvement, failed closure cases were excluded from postoperative VA analysis [18, 31–33]. The index showed significant correlation to postoperative VA in univariate and multivariate analysis. The prognostic value of MHRI was further evaluated by ROC curve analysis for visual success defined as postoperative VA ≤ 0.3 LogMAR. Our results showed that the AUROC for predicting visual success after FTMH surgery was 76.1%, with sensitivity and specificity of 65.4% and 89.4%, respectively. The cutoff value was < 1.38, showing a predicted LogMAR vision of 0.3 or better postoperatively. As MHRI is a ratio of the maximum central RPE reflectivity to the minimum paracentral RPE reflectivity, lower central reflectivity and higher paracentral reflectivity correspond to lower MHRI values. Although the maximum central reflectivity index (CRI-Max) did not show any statistically significant correlation to the visual outcome, higher reflectivity was associated with lower postoperative VA. Govetto et al. have described supra-RPE granular deposits, which are hyperreflective areas above the RPE at the base of the hole and were associated with worse postoperative VA [32]. They suggested that these deposits may be remnants of photoreceptor outer segments. We might assume that one of the reasons for higher central RPE reflectivity in macular holes may be these deposits. As these deposits induce RPE hyperreflectivity in OCT, they cause a higher MHRI ratio. Our results noted higher ratios in failed closures and those with worse final visual acuity, which may supposedly be an indicator of a more chronic macular hole. Additionally, the minimum paracentral reflectivity index (PCRI-Min) showed a negative correlation with postoperative LogMAR VA, which indicates that higher RPE reflectivity in the paracentral area of the macular hole correlated with better postoperative visual acuity. As a macular hole is being formed, its walls become edematous, parafoveal intraretinal pseudocysts appear, and the photoreceptor outer segments are separated from the RPE cells [34]. On OCT imaging, the edges of the macular hole become elevated; this presumably contributes to the paracentral hyporeflectivity of the RPE. This process leads to degeneration and loss of the photoreceptors, thus leading to less postoperative VA gain [23]. Several studies have shown that parafoveal intraretinal pseudocysts were correlated with low closure rate and lower postoperative VA [19, 24, 30]. In contrast, some other studies found that cystic edges were associated with anatomical closure and a better final VA [18, 20, 35]. Moreover, MHRI and MLD were positively correlated, and larger macular holes showed higher MHRI ratios. We propose that higher paracentral RPE reflectivity generally indicates relatively intact RPE and preserved photoreceptor–RPE alignment, whereas lower paracentral reflectivity may reflect photoreceptor loss, edema, or cystic disruption. In contrast, central RPE reflectivity may be elevated in more chronic and larger holes due to fibrosis or granular deposits. The combination of low paracentral and high central RPE reflectivity contributes to an increased MHRI, which is associated with poorer postoperative visual outcomes.

We acknowledge that the present study has several limitations. This study is a retrospective case series with a relatively small number of cases. No a priori sample-size calculation was performed due to the retrospective design of the study. In addition, the marking of OCT hypo- and hyperreflective sections is subjective, although the inter-rater agreement in our study was high. Another limitation of our study is that MHRI, being derived from OCT reflectivity, can be influenced by acquisition parameters, signal strength, and device-specific processing. However, since MHRI is computed as a ratio of reflectivity within each scan, it is expected to be minimally influenced by image acquisition parameters, although the potential effect of these factors cannot be excluded. Furthermore, other factors such as the macular hole duration and stage, which were not evaluated, may also affect the MHRI. Future larger prospective studies, ideally multicenter and incorporating multiple devices, are needed to validate and confirm the predictive value of MHRI and compare it to previous prognostic OCT parameters and indices.

## Conclusions

The MHRI, an index based on central and paracentral RPE reflectivity, may be a useful indicator for predicting anatomical and visual outcomes after macular hole surgery, as suggested by our analysis. Higher MHRI values could hypothetically reflect macular hole chronicity and be potentially associated with photoreceptor disruption and thus lower postoperative visual acuity. In our study, macular holes with a minimum diameter < 313 μm or MHRI < 1.38 showed a better visual prognosis. Both parameters demonstrated relatively high specificity and moderate sensitivity, but these findings should be interpreted cautiously given the sample size and study design and require validation in larger, multicenter studies.

## Data Availability

The data sets generated during and/or analyzed during the current study are available from the corresponding author on reasonable request.
